# An Entropy-Based Model for Basal Ganglia Dysfunctions in Movement Disorders

**DOI:** 10.1155/2013/742671

**Published:** 2013-05-16

**Authors:** Olivier Darbin, Daniel Dees, Anthony Martino, Elizabeth Adams, Dean Naritoku

**Affiliations:** ^1^Department of Neurology, University of South Alabama, 307 University Boulevard, Mobile, AL 36608, USA; ^2^Division of System Neurophysiology, National Institute for Physiological Sciences, Okazaki, Aichi, Japan; ^3^Department of Neurosurgery, University of South Alabama, Mobile, AL 36608, USA; ^4^Department of Speech Pathology & Audiology, University of South Alabama, Mobile, AL 36608, USA

## Abstract

During this last decade, nonlinear analyses have been used to characterize the irregularity that exists in the neuronal data stream of the basal ganglia. In comparison to linear parameters for disparity (i.e., rate, standard deviation, and oscillatory activities), nonlinear analyses focus on complex patterns that are composed of groups of interspike intervals with matching lengths but not necessarily contiguous in the data stream. In light of recent animal and clinical studies, we present a review and commentary on the basal ganglia neuronal entropy in the context of movement disorders.

## 1. Introduction

 Characterization of the neuronal data stream of basal ganglia neurons has been the foundation of most of the functional models for movement disorders. The divergences (or complementarities) between these models mostly result from the analytical strategy used to characterize and to model the data stream of the basal ganglia (BG) neurons. The analysis of the firing rate has forged the “rate hypothesis” while the frequency analysis has forged the “oscillatory model.” Briefly, the rate hypothesis and oscillatory model suggest that increasing activity and/or beta oscillations in the output nuclei of the BG (the globus pallidus internal (GPi)) reduces motor selection and leads to hypokinesia in Parkinsonism. 

 Since this last decade, different groups have integrated new mathematical tools to characterize and/or model the activity of the BG neurons including nonlinear analyses which describe complex patterns in the neuronal data stream. After reviewing the recent findings from the nonlinear analysis of the BG neurons, we present a review on the avenues and hypotheses brought by these newly integrated mathematical tools and their possible impacts on the next generation of functional models of the basal ganglia. 

## 2. Linear Model for Basal Ganglia and Movement Disorders

 The basal ganglia are part of corticocortical loops (via the thalamus) [1–7] and have connections with the brainstem [8]. The striatum (Str) and subthalamic nucleus (STN) are the main input nuclei of the basal ganglia and receive excitatory glutaminergic input from the cortex. At the striatal level, this glutaminergic drive exerts a tonic excitatory control on the striatal efferent neurons (medium spiny neurons (MSN)) [9, 10] which project, via two pathways, to the basal ganglia output nuclei (the internal segment of the pallidum (GPi) and the pars reticulata of the substantia nigra (SNr)). The “direct” pathway is a GABAergic monosynaptic projection to these output nuclei, while the “indirect” pathway is a GABAergic multisynaptic projection via the globus pallidus external. An additional cortico-STN-GPi pathway provides a “hyperdirect” excitatory drive from STN to the GPi [11]. Therefore, neuronal activities in the basal ganglia output nuclei are dependent on the synergic activity between these pathways [12]. 

 The sequential and convergent arrangement of excitatory and inhibitory neurons in these nuclei has forged the concept that basal ganglia and, inherently, information processing rely on the summation of excitatory and inhibitory inputs and are therefore linear in nature. To compare neuronal activity in the basal ganglia to the model predictions, the measurements of the firing rate and other linear markers in the time domain have been examined in animal and clinical studies. Data from these studies have contributed to the “rate model” for movement disorders. This model is founded upon the assumption that the direct pathway (Str-GPi/SNr) is up-regulated by the D1 dopaminergic receptor and indirect pathway (Str-GPe-GPi/SNr) is down-regulated by the D2 dopaminergic receptor. The dynamic balance between these two pathways contributes in motor selection and motor inhibition, respectively [13]. The “rate model” predicts that dopamine depletion leads to an imbalance in favor of the indirect pathway resulting in increased activity in GPi and excessive motor inhibition in Parkinsonism [14–22]. In dystonia, the model predicts a decreased activity in the GPi and increased selection of motor programs [23, 24]. In contrast to a large body of experiments in animal models that favor the rate hypothesis, measurement of neuronal firing rates in the basal ganglia of patients with hypokinesia or hyperkinesia has given conflicting data with some studies supporting [25–31] and others not supporting the rate hypothesis [32–36]. 

In addition to time domain analyses which are based on the probability distribution of interspike intervals (ISIs), other studies have characterized the firing activity of basal ganglia neurons in the frequency domain. In a majority of studies, differences in oscillatory activities have been identified between normal and pathological conditions [37–42]. In PD patients, oscillations in the beta range of 11–30 Hz have been reported to occur in approximately 30% of GPi neurons, while, in dystonia patients, lower beta frequencies in the 8–20 Hz are dominant but present in approximately 10% of GPi neurons [43]. 

## 3. Evidence for Nonlinear Dynamic in the Neuronal Data Stream of BG Neurons 

The linear analyses used to characterize the basal ganglia activities in time and frequency domains measure the resultant linear combinations of independent patterns in the data stream. These analyses characterize the interspike interval (ISI) series by the summation of probability distributions for different durations of ISIs (i.e., firing rate, its range, or standard deviation) or several frequencies (power spectrum). However, the irregularity in the neuronal firing activity is not linear [44–51] since complex patterns can occur more often than predicted due to the probability distribution of the ISI series. In the last decade there has been a growing body of evidence that linear analysis does not fully describe the activity of neuronal networks and has justified the use of nonlinear analyses to further characterize the ISIs series [52–57]. These studies have identified neuronal patterns composed of either nonadjacent ISIs occurring nonperiodically, or patterns similar in shape but repeatedly occurring in different time scale (or size). The term “nonlinear temporal organization” has been introduced to define patterns identified by nonlinear analyses in the neuronal data stream. An initial finding by our group is that nonlinear temporal organization is present in a series of consecutive ISIs recorded from basal ganglia neurons in the awake normal primate [55] and in Parkinsonian patients [57]. These analyses have established that the temporal organization of ISIs in the time series results in the replication of complex patterns that cannot be statistically explained by random trials from the probability distribution of the ISIs [55–57].

## 4. Sensitivity of Nonlinear Markers Regarding Pathological Conditions

The clinical relevance of these nonlinear features in neuronal discharge is not yet clear. Specifically, it is unknown whether the nonlinear dynamics of basal ganglia neurons are affected by the conditions of parkinsonism or dystonia. In retrospective analyses of a database of PD and dystonia neurons with temporal organizations (as defined in [55]), Sanghera et al. [58] found a higher neuronal entropy (as estimated by the Approximate Entropy (ApEn) [59]) in the GPi of PD patients comparatively to dystonia patients. Currently, a major difficulty in the interpretation of these data, regarding the effects of the disorder per se, is the lack of comparison to the normal state of the basal ganglia. Nevertheless, these last data, in line with the decreases in neuronal entropy observed following deep brain stimulation (DBS) treatment in animal models for Parkinsonism [52] or apomorphine administration in PD patients [56], suggest that high neuronal entropy (at least in the GPi) is associated to lower kinetic activity. It is worth noting that, by construction, Approximate Entropy (ApEn) remains unchanged under uniform process magnification, reduction, and translation [59]. Therefore, changes in overall firing rate (per se) cannot explain the changes in neuronal entropy reported following treatments or between disorders.

Pharmacological studies in primate models for movement disorders are needed to further investigate this hypothesis as their basal ganglia neuronal activity exhibits similar patterns to those seen in patients [14]. In addition, it would be relevant to investigate whether similar markers can be identified in global signals, such as the EEG, which could provide a strategy to compare healthy condition to conditions with movement disorders in humans. 

## 5. The “Entropy Hypothesis”

Since anti-Parkinsonian treatments decrease entropy and hyperkinetic conditions are associated with lower entropy, it is time to include the basal ganglia neuronal entropy as a putative interfering factor in the current model for the selection and the inhibition of motor information in the basal ganglia circuitry. Through exploring the current data framework available, we present a primary hypothesis on the nature of the GPi neuronal entropy regarding abnormal movement production.

**Figure 1:**
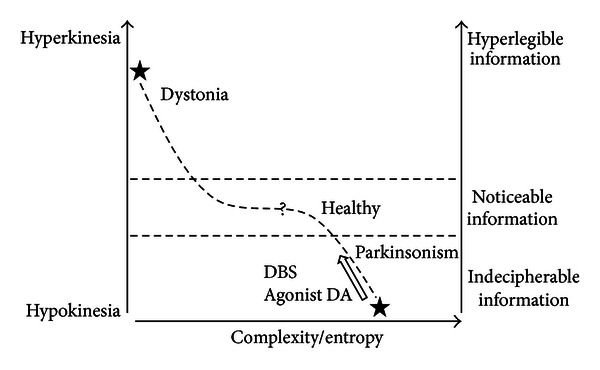
This figure shows a hypothetical relation between neuronal entropy and the aptitude of the downstream of information to generate movement regarding the conditions of hypokinesia and hyperkinesia. Low neuronal complexity could result in a hyperlegible signals and increased motor production. In contrast, high neuronal complexity could result in an indecipherable signal reducing motor production. Anti-Parkinsonian treatments reduce both hypokinesia and complexity. Since the comparisons between movement disorders and control remain unestablished, the healthy condition is envisaged with an adequate degree of GPi complexity allowing the emergence of noticeable information related to voluntary movement.

From the data discussed above, we hypothesize that high entropy in the GPi neuronal data stream is associated to an increased motor inhibition (i.e., parkinsonism) while reduced entropy in the GPi neuronal data stream is envisaged as a feature for increasing motor selection (i.e., dystonia or anti-Parkinsonism treatments) (see Figure 1). This hypothesis underlies the idea that high entropy in the GPi neuronal data stream would correspond to a network condition generating a large number of different pattern possibilities leading to a signal with limited order or “organization” and reduced information. Therefore, the higher GPi neuronal entropy reported in parkinsonism can be conceptualized under Shannon-Brillouin's interpretation [60] as a measure of disorder, unpredictability, and reduced motor information leading to hypokinesia. This hypothesis is not opposed to the current model of selection and inhibition of motor programs along the basal ganglia circuitry [11] but introduces the GPi neuronal complexity as a factor which enhances the inhibition of motor program by decreasing the informative nature of the neuronal data stream. The “entropy hypothesis” predicts that lower entropy would increase information in the data stream and motor program selection resulting in hyperkinesia. 

## 6. Biological Substrata for the “Entropy Hypothesis”

The relation between the entropy theory and the functions of the basal ganglia can be substantiated by the intrinsic (and logarithmic) relation between entropy and the correlation dimension [61]. Since the correlation dimension is a measure of the dimensionality of the space occupied by a data series, neuronal entropy can be envisaged as a related measurement of dimensionality of the neuronal data stream. The concept that correlated information from large population of neurons can be compressed into a selected number of neurons has been suggested to be an important mechanism for information encoding in the brain [62] and the basal ganglia especially [63]. High GPi neuronal entropy can underlie an inadequate compression (or reduction of the dimensionality [64]) of upstream population activity into the output neurons of the BG circuitry [63]. Alternatively, some circuitry reorganization such as increased interconnections between elements of the motor circuitry [65] may have the potential to increase the correlation dimension of the stream of information to the GPi neurons. Experimental research in animal models for movement disorders is warranted to explore these avenues.

## 7. Conclusion

The use of nonlinear domain analyses to describe the neuronal and network activities inside the basal ganglia may provide new qualitative and quantitative information regarding the nature of the sensory-motor processing as well as its distortion in pathological conditions. It is expected that the inclusion of key nonlinear features into silicone-based models of the basal ganglia could better reproduce complex and nonstationary signals recorded in normal and pathological conditions. To date, the “entropy hypothesis” may be useful to initiate a debate on nonlinear dynamics in basal ganglia activity and their roles in the selection and inhibition of motor programs. Most importantly, these nonlinear analyses may contribute to reduce the gap between the basal ganglia models and the theories on the processing of motor information.
